# Coherent Phonon Transport Measurement and Controlled Acoustic Excitations Using Tunable Acoustic Phonon Source in GHz-sub THz Range with Variable Bandwidth

**DOI:** 10.1038/s41598-018-25525-2

**Published:** 2018-05-04

**Authors:** Xiaohan Shen, Zonghuan Lu, Yukta P. Timalsina, Toh-Ming Lu, Morris Washington, Masashi Yamaguchi

**Affiliations:** 1 Center for Materials, Devices, and Integrated Systems, and Department of Physics, Applied Physics and Astronomy, Rensselaer Polytechnic Institute, Troy, New York, 12180 United States; 2 Jiangsu Hengtong Optical Network Technology Co., Ltd., Suzhou, Jiangsu Province 215200 China

## Abstract

We experimentally demonstrated a narrowband acoustic phonon source with simultaneous tunabilities of the centre frequency and the spectral bandwidth in the GHz-sub THz frequency range based on photoacoustic excitation using intensity-modulated optical pulses. The centre frequency and bandwidth are tunable from 65 to 381 GHz and 17 to 73 GHz, respectively. The dispersion of the sound velocity and the attenuation of acoustic phonons in silicon dioxide (SiO_2_) and indium tin oxide (ITO) thin films were investigated using the acoustic phonon source. The sound velocities of SiO_2_ and ITO films were frequency-independent in the measured frequency range. On the other hand, the phonon attenuations of both of SiO_2_ and ITO films showed quadratic frequency dependences, and polycrystalline ITO showed several times larger attenuation than those in amorphous SiO_2_. In addition, the selective excitation of mechanical resonance modes was demonstrated in nanoscale tungsten (W) film using acoustic pulses with various centre frequencies and spectral widths.

## Introduction

Accurate determination of frequency-dependent acoustic phonon transport properties in the GHz to sub-THz frequency range is critical to in-depth comprehension of nanomechanical properties^1–9^ and control of structural dynamics^10–15^ in nanoscale materials. Characteristic length scale of many nanoscale materials is comparable to the acoustic phonon wavelength in that frequency range. The picosecond ultrasonic technique, which excites broadband acoustic pulses with a spectral bandwidth of hundreds of GHz^16,17^, has been used in a number of applications. For narrowband pulses, Sun *et al*.^18^ and Lin *et al*.^19^ have reported the generation of multiple-cycle acoustic phonons in the GHz to sub-THz frequency range using InGaN/GaN multiple quantum wells, where coherent acoustic phonons were excited using the sudden screening of the strain induced piezoelectric field by the photo-generated carriers. However, this method requires a permanent quantum well structure for a particular frequency, and the multiple samples are required for frequency-dependent measurements. Choi *et al*.^20^ and Klieber *et al*.^21^ proposed a method for optical generation of frequency tunable, multiple-cycle acoustic phonons in the 20 to 400 GHz frequency range using a retroreflection-based optical pulse shaper, “Deathstar”. In their experimental setup, a single ultrafast optical pulse was split into seven pulses to form a pulse train with Gaussian envelope. The excited acoustic phonon frequency was controlled by the time separation of the optical excitation pulses, while the acoustic phonon spectral bandwidth was constant due to the fixed number of optical pulses. Complete control of the centre frequency and spectral bandwidth of acoustic pulse shape are advantageous for the selective excitation and the precise measurements of nanoscale mechanical properties.

In the present work, we demonstrate the generation of controlled quasi-monochromatic coherent acoustic phonons with tunable centre frequencies and spectral bandwidths, and its applications to the study of acoustic transport experiments in the GHz to sub-THz frequency range. The simultaneous tunings of the centre frequency and the bandwidth of the acoustic pulses are achieved by shaping optical excitation pulses for the generation of the acoustic pulses with desirable character. The intensity modulated optical excitation pulses were generated by the interference of two chirped optical pulses^22^. The centre frequency of the acoustic phonons can be tuned by changing the time delay between the two chirped optical pulses. The spectral bandwidth tuning of the acoustic phonons was achieved by adjusting the pulse widths of chirped optical pulses, hence the number of cycles within the coherent acoustic pulse. Here, we demonstrate the applications of this technique for the determination of the frequency-dependent acoustic attenuation and sound velocity in SiO_2_ and ITO films and the selective excitation of mechanical resonance modes in W thin film.

## Results

### The scheme of spectrum-tunable narrowband acoustic phonon spectroscopy

**Figure 1:**
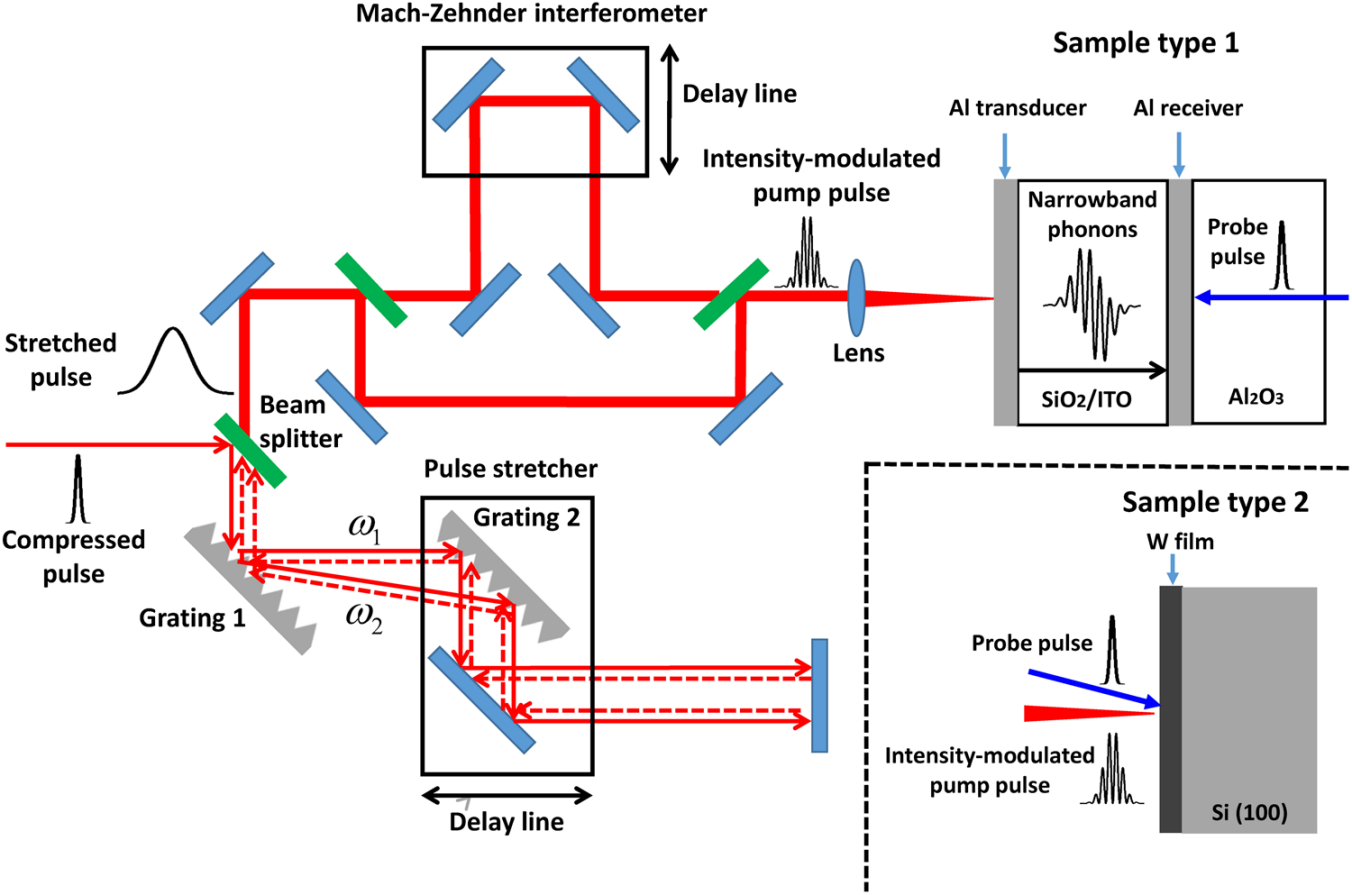
The scheme of spectrum-tunable narrowband acoustic phonon spectroscopy. Schematics of the experimental setup of the frequency and bandwidth tunable narrowband acoustic phonon spectroscopy using the intensity-modulated optical pump pulse. The sandwiched-layer sample (sample type 1) was used for the acoustic transport measurement by the opposite-side pump-probe geometry. The nanoscale W thin film (sample type 2) was used for the study of mechanical resonant eigen-mode excitation by the same-side pump-probe geometry.

The schematic of our experimental setup is shown in Fig. 1. The electrical field of linearly chirped Gaussian pulse after passing through the pulse stretcher is given by^22^

1 $$E(t)\cong {E}_{0}\sqrt{\frac{\sigma }{{\sigma }_{n}}}{\exp }\{-\frac{{t}^{2}}{{\sigma }_{n}^{2}}[1-i(\frac{\sigma }{{\sigma }_{n}})]+i[{\varphi }_{0}-{\omega }_{0}(t+{\tau }_{0})-\frac{\pi }{4}]\},$$

where *E*_0_, *t*, *ω*_0_, *τ*_0_, and *ϕ*_0_ are the electrical field amplitude of linearly chirped Gaussian pulse, time, carrier angular frequency of compressed optical pulse, the group delay at *ω*_0_ and the initial phase, respectively. *σ* and *σ*_*n*_ is the *e*^−1^ half-width of the compressed optical pulse and the linearly chirped optical pulse, respectively. The intensity of the recombined output optical pulses after passing through the Mach-Zehnder interferometer can be written as^22^

2 $$I(t)=\frac{1}{2}{I}^{+}(t)+\frac{1}{2}{I}^{-}(t)+{E}_{0}^{2}(\frac{\sigma }{{\sigma }_{n}}){\exp }[-(\frac{2{t}^{2}}{{\sigma }_{n}^{2}}+\frac{{\tau }^{2}}{2{\sigma }_{n}^{2}})]{\cos }(\frac{2t\tau }{{\sigma }_{n}\sigma }-{\omega }_{0}\tau ),$$

${I}^{+}(t)\,$and ${I}^{-}(t)$ are DC components of optical pump pulse intensity, and given by ${I}^{\pm }(t)={E}_{0}^{2}(\frac{\sigma }{2{\sigma }_{n}}){\exp }[-\frac{2{(t\pm \tau /2)}^{2}}{{\sigma }_{n}^{2}}]$. The third term is the quasi-sinusoidal optical modulation at the beat frequency, ${f}_{0}(\tau ,{\sigma }_{n})=\frac{\tau }{\pi \sigma {\sigma }_{n}}$, where *τ* is the time delay between the two optical pulses. The generated intensity-modulated optical pulse was further used to excite a metallic transducer to generate the acoustic phonons through the photothermal effect^16,23^. The generated mechanical strain due to the effect is proportional to the intensity of the optical excitation pulse, and therefore, the acoustic phonons excited by the intensity-modulated optical pulse were quasi-monochromatic.

### Model of coherent phonons excited by intensity-modulated optical pulse

Coherent acoustic signals are calculated based on a simulation model as discussed in the following section. The lattice temperature *T* after the optical pulse excitation is governed by the thermal diffusion equation^24^

3 $$C\frac{\partial T}{\partial t}=\frac{\partial }{\partial z}(\kappa \frac{\partial T}{\partial z})+\alpha (1-R)I(t){e}^{(-\frac{z}{l})},$$

where *C* is the lattice heat capacity per unit volume, *κ* is the thermal conductivity, *l* is the optical penetration depth of pump pulse, *R* is the reflectance of the optical pump pulse, and *I(t)* is the temporal profile of the intensity-modulated optical pump pulse as described in Eq. (2). The wave equation of longitudinal acoustic phonons induced by the thermal expansion is given by^24,25^

4 $$\rho \frac{{\partial }^{2}u}{\partial {t}^{2}}=\frac{\partial {\sigma }_{33}}{\partial z}=\rho {v}^{2}\frac{{\partial }^{2}u}{\partial {z}^{2}}-\frac{\partial G}{\partial z}-\Gamma \frac{\partial u}{\partial t},$$

where *u* is the mechanical displacement, *ρ* is the mass density, *σ*_33_ is the longitudinal stress, *v* is the longitudinal acoustic velocity, *G* is the potential of the force field^24^, *Г* is the linewidth associated with the acoustic damping^26^. In the case of thermoelastic generation with the electrons and lattice that are in thermal equilibrium, the potential of force field can be written as $G=\gamma C\delta T$^24^, where *γ* is the Grüneisen parameter. The lattice temperature rise is given by $\delta T=T-{T}_{0}$, where *T* is the lattice temperature after optical excitation, which is calculated using Eq. (3), and *T*_0_ is the initial lattice temperature.

### Experimental configuration

Two types of experimental configurations are shown in Fig. 1. The first type of the configuration is for the frequency-dependent acoustic phonon transport experiment, and the second type of the configuration is for the mechanical excitation of nanoscale materials. In the acoustic phonon transport experiment, the sample has the sandwiched-layer structure consisting of an aluminium (Al) transducer film, a sample layer (SiO_2_ or ITO), and an Al receiver film deposited on bulk sapphire substrate (sample type 1). The thicknesses of the Al transducer and receiver films were 10 nm and 50 nm, respectively. The Al transducer film was excited with the intensity-modulated optical pump pulse and quasi-monochromatic acoustic phonons were generated through the photothermal effect^16,23^. The optically generated acoustic phonons propagated through the sample layer and were detected at the Al receiver film by an optical probe pulse through the photoelastic effect^16,23^. The optical pump power was 3 mW and the optical probe power was 5 μW. The optical pump and probe pulses were focused onto the sample surface by optical lens, and the diameters were 100 μm and 20 μm, respectively. The thickness of the Al receiver film was chosen to be larger than the penetration depth of optical probe beam to block the probe transmission through the receiver film. Two sample sets with sample layer thicknesses of 20 nm and 218 nm were used for the determination of sound speed and acoustic attenuation.

In the experiment of the mechanical excitation of thin film, a 15 nm thick W film deposited on bulk Si (100) substrate (sample type 2) was used as a sample. The optical pump pulse was normal incident on the surface of W film to excite the nanomechanical resonance of the film, and the probe pulse was sent to the same side of W film with 15 degree away from normal incidence to detect the acoustic phonons reflected at the interface between W film and Si substrate.

### Narrowband acoustic phonon source with frequency tunability

**Figure 2:**
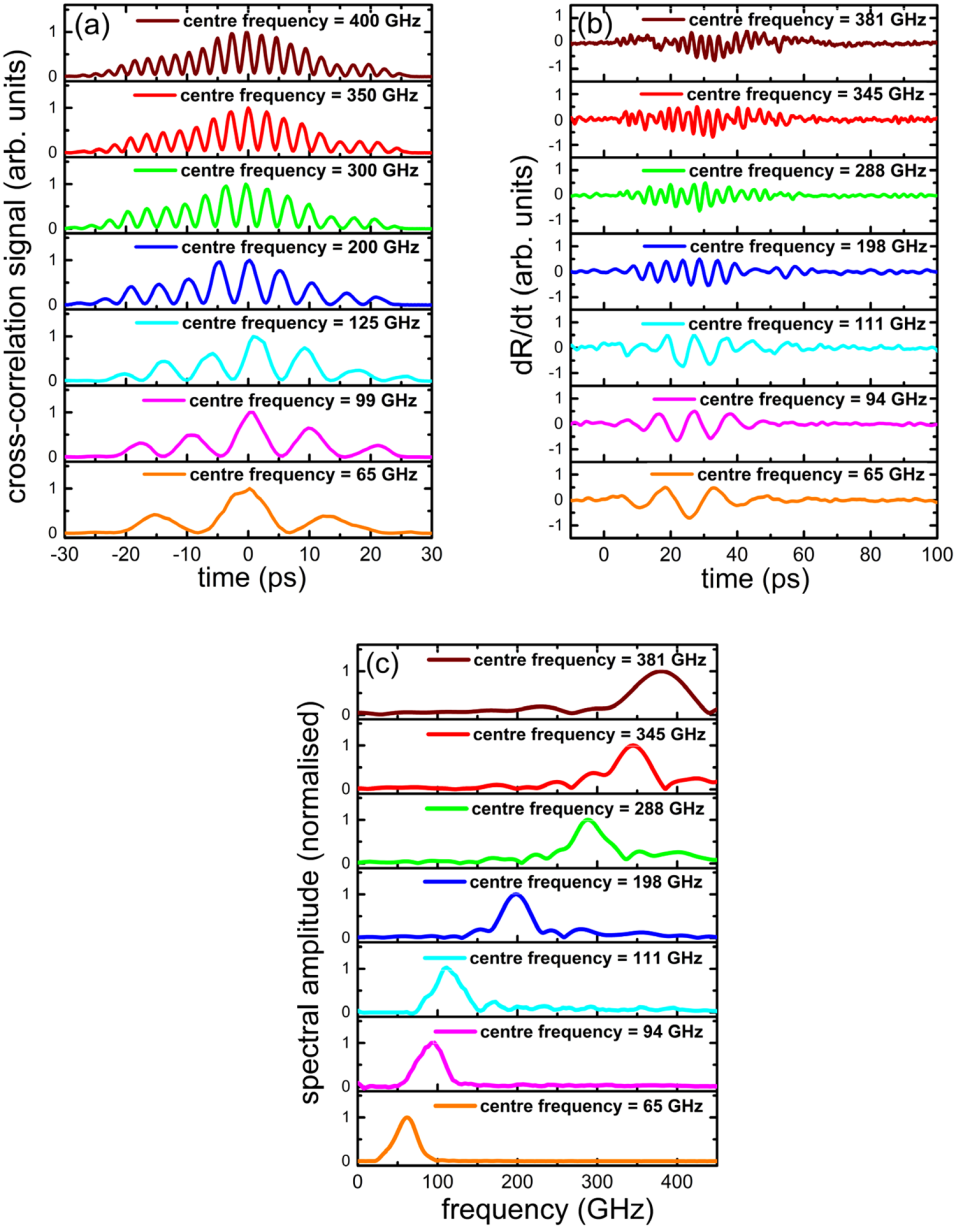
Demonstration of narrowband acoustic phonon source with frequency tunability. (**a**) The temporal profiles of the optical excitation pulses, measured as the cross-correlation signals of the intensity-modulated optical pump pulse and a 100 fs optical pulse, at various centre frequencies from 65 to 400 GHz. (**b**) The differential transient reflectivity signals induced by narrowband acoustic phonons. (**c**) The Fourier spectrums of the corresponding transient reflectivity signals.

Figure 2(a) shows the cross-correlation signals of the intensity-modulated optical pump pulse and a compressed optical pulse with the pulse width of 100 fs to represent the temporal profiles of the optical excitation pulses. The centre frequency of the intensity-modulated optical pulses can be tuned from 50 GHz to 2 THz, the tuning range was limited by the maximum and minimum relative delays between the two paths in the current experimental setup. However, the maximum acoustic phonon frequency in the current experiment was limited to 400 GHz by the bandwidth of the Al transducer. The chirped pulse width was kept at 23 picoseconds (ps), and the time delay between two chirped pulses was tuned from 0.4 to 2.4 ps. Correspondingly, the modulation frequency of the intensity-modulated optical pulse was changed from 65 to 400 GHz. The number of the oscillations within the excitation pulses increases as the centre frequency increases, hence the spectral bandwidth becomes narrower. The intensity-modulated optical pulse was then used as the pump pulse for the generation of quasi-monochromatic acoustic phonons in the Al transducer via photothermal effect. Figure 2(b) shows the differential transient reflectivity signals induced by the generated narrowband acoustic phonons. A major part of the unwanted thermal background was eliminated by probing the acoustic signals from the receiver side, as thermal diffusion is low in SiO_2_. The differential transient reflectivity signal is shown in the Fig. (2b), where the residual thermal background contribution has been further eliminated. The corresponding Fourier spectra show the continuous tunability of the centre frequency of the excited acoustic pulses in Fig. (2c). The centre frequency was tuned from 65 to 381 GHz, and the spectral bandwidth was fixed to 30 GHz.

### Narrowband acoustic phonon source with bandwidth tunability

**Figure 3:**
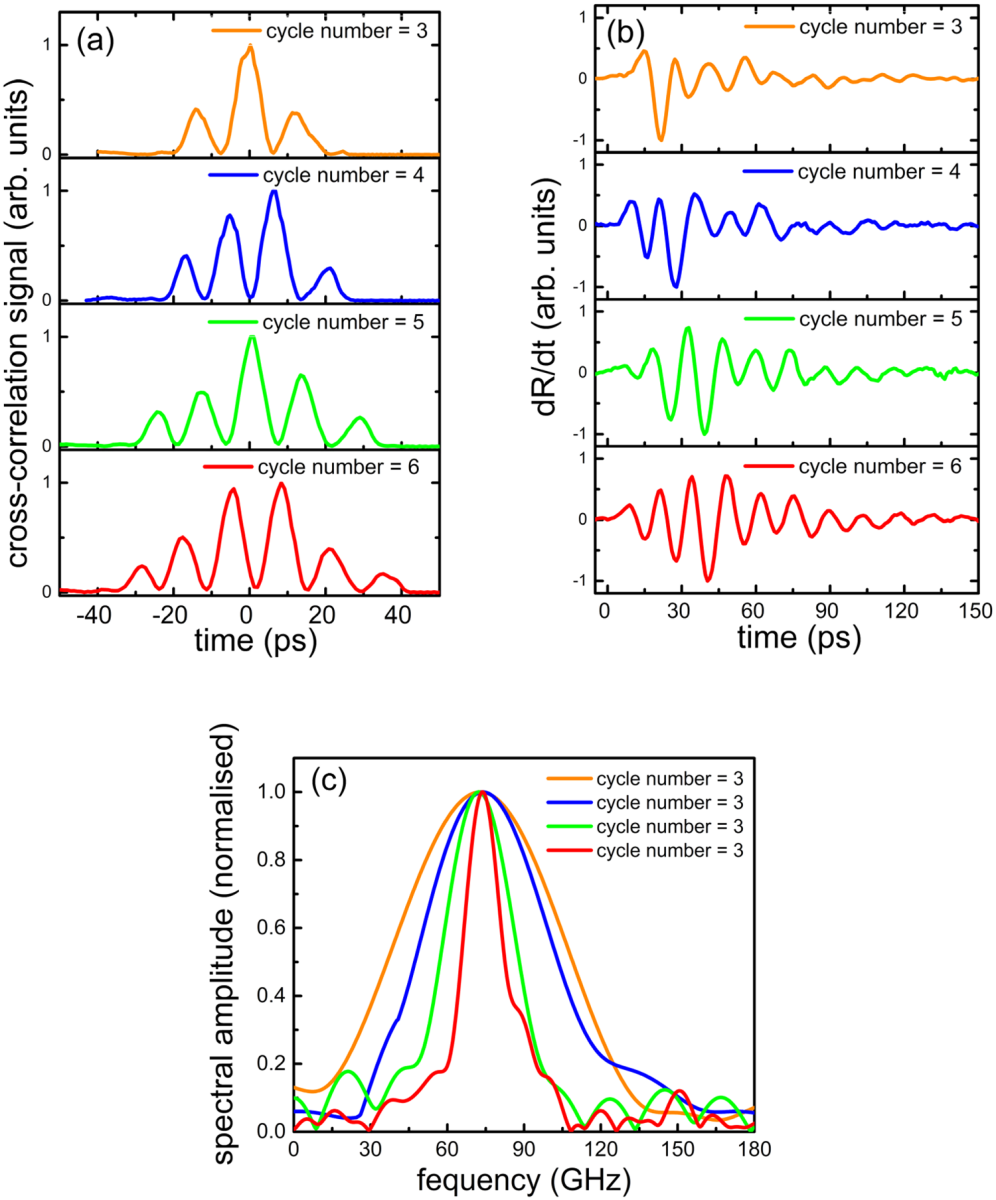
Demonstration of narrowband acoustic phonon source with bandwidth tunability. (**a**) Cross-correlation signals of the intensity-modulated optical pump pulse with different numbers of cycle of oscillation, and a 100 fs optical pulse. (**b**) The differential transient reflectivity signals induced by the narrowband acoustic phonons after optical excitation. (**c**) The Fourier spectra of the corresponding transient reflectivity signals in (**b**).

Figure 3 shows the spectral bandwidth tunability of the excited acoustic pulse. The sample involved in the experiment had a sandwiched layer structure consisting of an Al transducer layer, a SiO_2_ layer and an Al receiver layer. The temporal profiles of the intensity-modulated optical pump pulses are shown in Fig. (3a), the number of the oscillations within the pulse was varied while the centre frequency was kept constant. The number of the oscillations is proportional to the chirped optical pulse width *σ*_*n*_, while the centre frequency is proportional to $\frac{\tau }{{\sigma }_{n}}$. By adjusting translational stage positions for *σ*_*n*_ and *τ*, tunings of the centre frequency and the bandwidth of the acoustic pulses are achieved. In this experiment, the pulse width of the chirped optical pulses was tuned from 15 to 25 ps, and the time-delay between the two chirped optical pulses was tuned from 0.4 to 0.7 ps. The acoustic phonon signals with various bandwidths is shown in Fig. (3b). The corresponding Fourier spectra of the transmission signals are shown in Fig. (3c), where the spectral bandwidth became narrower as the number of the oscillations increased. The spectral bandwidths of the quasi-monochromatic acoustic phonons were changed from 17 to 73 GHz, while the centre frequency was kept at 75 GHz. The minimum spectral bandwidth of 17 GHz is about 20 times narrower than the spectral bandwidth of the broadband acoustic phonons^17^ excited by a single compressed ultrafast optical pulse.

### Control of acoustic excitation experiment

**Figure 4:**
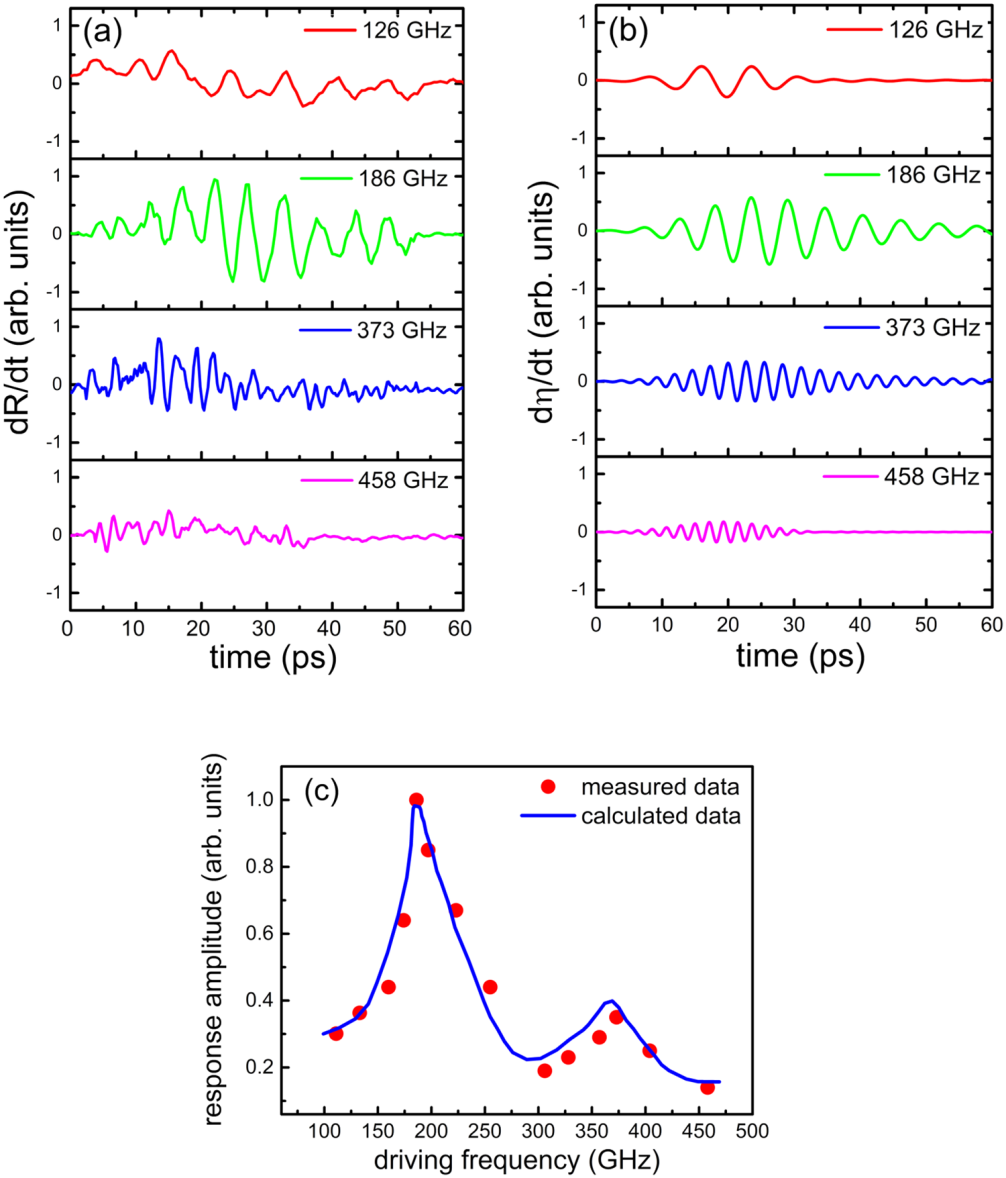
Acoustic excitation of mechanical resonance modes of W film. (**a**) The measured signals of differential transient reflectivity from W film induced by narrowband acoustic phonons at various driving frequencies from 126 to 458 GHz. (**b**) The calculated signals of differential strain on the surface of W film at corresponding driving frequencies. (**c**) The amplitude of the acoustic response in W film as a function of driving frequency, red dots are measured data and blue curve is calculated data.

The frequency-dependent acoustic response of a 15 nm thick W film is shown in Fig. (4a). W has been chosen because of higher bulk modulus than Al. Experimental configuration of type 2 in Fig. 1 was used. Further, the frequency profile of the acoustic signal was numerically simulated by calculating the strain propagation inside the W film for the comparison to the experimental data in Fig. (4b). The acoustic reflection coefficients at the interfaces were determined using the acoustic mismatch model (AMM)^27^, expressed as $r=\frac{{Z}_{1}-{Z}_{2}}{{Z}_{1}+{Z}_{2}}$, where *Z*_1_ is the acoustic impedance of the material that acoustic wave originally propagates and *Z*_2_ is the acoustic impedance of the second material. The acoustic impedance of material is given by $Z=\rho v$, where *ρ* is the mass density and *v* is the sound velocity. Following parameters were used for the calculation: the longitudinal acoustic velocities of W and Si were 5300 m/s and 8445 m/s respectively^28,29^, and mass densities of W and Si were 19250 kg/m^3^ and 2330 kg/m^3^ respectively^30^. The displacement of W film was calculated by numerically solving Eqs (2), (3) and (4) along with the boundary conditions, using the finite difference time domain (FDTD) analysis. The strain of the W film was calculated by the derivative of displacement as $\eta =\partial u/\partial z$. The reflectivity change *ΔR* is induced when the strain pulse reaches to the film surface, where *ΔR* is proportional to the strain amplitude *η*^16^. Hence, $dR/dt\propto d\eta /dt$. The calculated signals of $d\eta /dt$ detected on the surface of W film are shown in Fig. (4b), which are proportional to the measured signals of $dR/dt$. The experimental data shows the coherent oscillations, and the signal amplitude depends on the excitation frequencies.

Figure (4c) shows the amplitudes of the experimental and simulated signals as a function of driving frequency. Simulated signals are fitted to the experimental data with the film thickness and the damping constants as fitting parameters. Measured and simulated signals indicate good agreement, and show two peaks at the frequencies of 186 GHz and 372 GHz. The second frequency is the second harmonics of the first frequency, and these peaks are assigned to the first and second longitudinal mechanical resonance modes in the W film.

The linewidth extracted from the best fitting results was *Г* = 10 GHz. The acoustic attenuation *α* was related to the linewidth by the formula $\Gamma =\alpha v\,$^26^, where *v* is the sound speed. The corresponding acoustic attenuation of W film was *α* = 1.89 × 10^6^ m^−1^, which was in the same order compared to the acoustic attenuation of vitreous silica, i.e. 1 × 10^6^ ~ 8 × 10^6^ m^−1^, in the same frequency regime (100 ~ 400 GHz)^21,26^. The amplitude of the first resonance peak was observed to be larger than the second resonance peak.

The thickness of the W film was determined as 14.2 nm by using the simple equation for the mechanical resonance, ${f}_{n}=n\frac{{v}_{l}}{2d}$ (*n* = 1, 2, 3…)^31^ with the literature value of the sound velocity of W, ${v}_{l}=5300\,m/s\,$^28^, whereas the direct measurement of the film thickness by scanning electron microscope (SEM) shows 15 nm. The discrepancy of 0.8 nm is within the surface roughness of the film of 1 nm, which was determined by SEM.

The selective excitation of the nanomechanical longitudinal resonance modes in metallic W thin film was demonstrated in GHz-sub THz frequency range using the narrowband acoustic phonon source. It has potential applications in control of acoustic/mechanical resonance modes in nanoscale materials.

### Frequency-dependent acoustic phonon transport experiment

**Figure 5:**
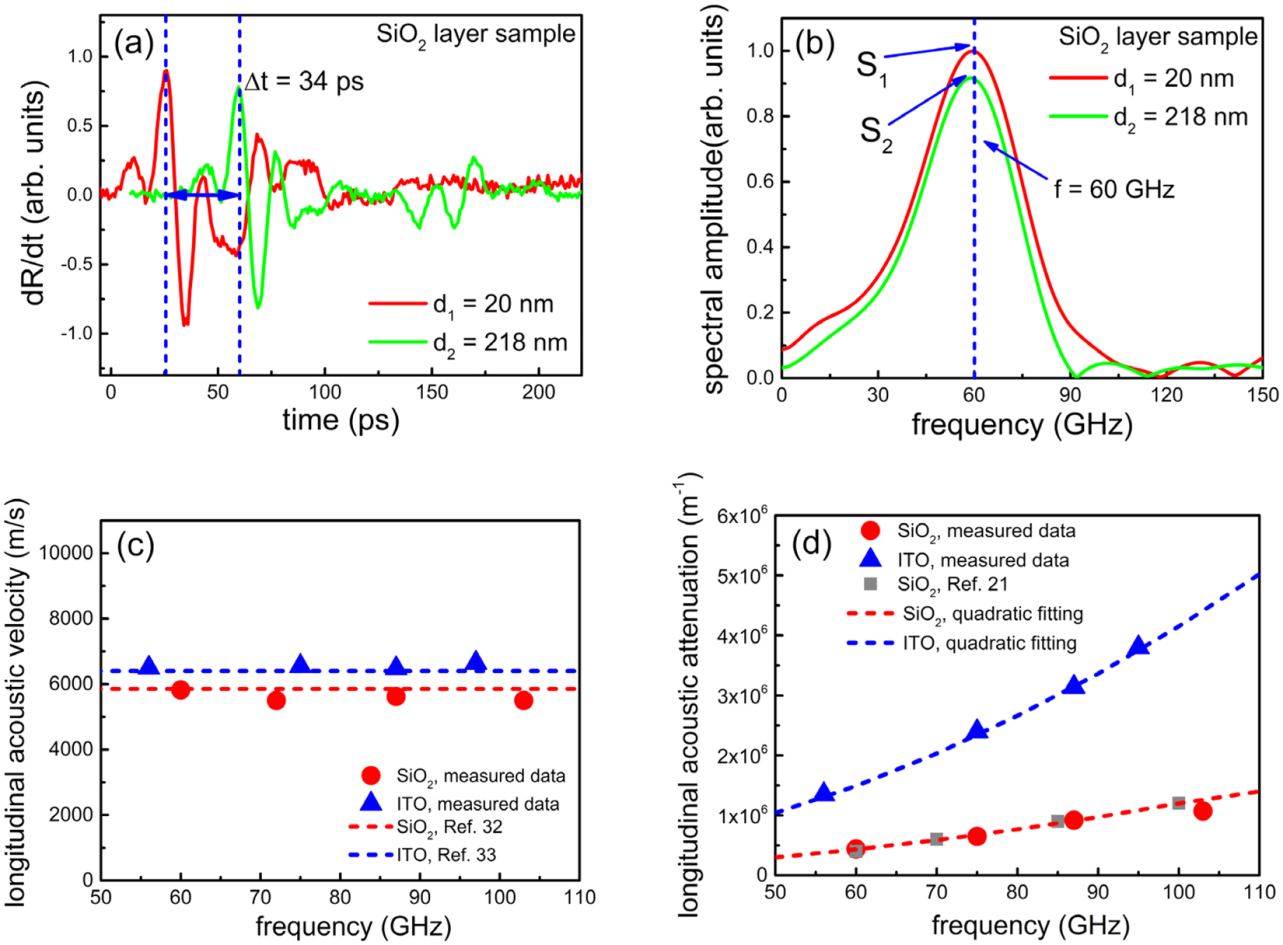
Measurement of frequency-dependent acoustic phonon transport in SiO_2_ and ITO films. (**a**) The transient reflectivity signals induced by narrowband acoustic phonons with centre frequency at 60 GHz transmitted through SiO_2_ layers of different thicknesses, i.e., 20 nm and 218 nm. (**b**) The Fourier spectrums of the corresponding transmitted acoustic phonon signals at frequency of 60 GHz. (**c**) The longitudinal acoustic phonon velocity of SiO_2_ and ITO at various frequencies from 50 to 110 GHz. (**d**) The longitudinal acoustic phonon attenuation coefficients of SiO_2_ and ITO at various frequencies from 50 to 110 GHz.

The frequency tunable narrowband acoustic phonon source was further applied to the measurements of the frequency-dependent acoustic phonon transport in SiO_2_ and ITO layers using type 1 sample in Fig. 1. SiO_2_ is known to have a large dispersion of attenuation while the direct measurements are still rare in this frequency range^20,21^. ITO is polycrystalline material with free carriers, and strong phonon attenuation is expected. The signals from the acoustic phonons at 60 GHz which were transmitted through SiO_2_ layers with different thicknesses of ${d}_{1}=20\,nm$ and ${d}_{2}=218\,nm$, are shown in Fig. (5a). The acoustic phonons were excited from one side of the sample and were detected from the other side of the sample as illustrated in Fig. 1.

The acoustic phonon signal appeared on the receiver side after the phonons propagated through the SiO_2_ layer. The difference of Time-of-Flight (TOF) between the two samples with different thicknesses of SiO_2_ layer was $\Delta t=34\,ps$. The longitudinal acoustic phonon velocity in SiO_2_ layer was determined by ${v}_{l}={\rm{\Delta }}d/{\rm{\Delta }}t$, where $\Delta d=198\,nm$ was the thickness difference of two SiO_2_ samples. Moreover, the amplitude of the acoustic phonon signal decreased as it transmitted through the thicker SiO_2_ layer due to the attenuation in the SiO_2_ layer. Figure (5b) illustrates the corresponding Fourier spectra of the transmitted acoustic phonon signals at 60 GHz, where the spectral amplitude ${S}_{2}(\omega )$ of 218 nm thick SiO_2_ sample was smaller than the spectral amplitude ${S}_{1}(\omega )$ of 20 nm thick SiO_2_ sample, which was attributed to the acoustic attenuation. The Fourier amplitudes ${S}_{1}(\omega )$ and ${S}_{2}(\omega )$ were related as ${S}_{2}(\omega )={e}^{ik(\omega )\Delta d}{S}_{1}(\omega )$, where $k(\omega )$ is the complex wave vector whose imaginary portion determined the frequency-dependent acoustic attenuation coefficient $\alpha (\omega )$, given by^20,21^

5 $$\alpha (\omega )=\frac{1}{{\Delta }d}ln\frac{{S}_{1}(\omega )}{{S}_{2}(\omega )}.$$

Figure (5c) shows the measured values of the longitudinal acoustic phonon velocity of SiO_2_ and ITO layers at different frequencies in the range of 50 to 110 GHz, which were independent of the phonon frequency within the experimental error. The measured values of the speed of sound of SiO_2_ and ITO are 5800 m/s and 6500 m/s respectively, which were in good agreement with the literature values of bulk fused silica^32^ and ITO film^33^ in the frequency range of 50 to 110 GHz. Figure (5d) shows the measured longitudinal acoustic phonon attenuation coefficients of SiO_2_ and ITO films at various frequencies in the range of 50 to 110 GHz. The acoustic attenuation coefficients of both SiO_2_ and ITO increased as the frequency increased. The attenuation results for SiO_2_ were in good agreement with the values reported by Klieber *et al*.^21^. The acoustic attenuation coefficients of both SiO_2_ and ITO showed quadratic frequency dependence, i.e., $\alpha (\omega )\propto {\omega }^{2}$, which indicates that the acoustic attenuation mechanism of both SiO_2_ and ITO in the 50 to 110 GHz frequency range was dominated by the anharmonic interaction of acoustic waves with the thermal phonon bath^21,34^. In addition, the longitudinal acoustic attenuation coefficients of ITO were larger than SiO_2_ in the frequency range. SiO_2_ has amorphous structure and is known to have a low thermal conductivity, while ITO has cubic crystal structure with grain structure. The reason for the higher acoustic attenuation for ITO is not clear at this moment. A possible explanation is that the phonon scattering with the ionised impurities in ITO^33^ is more intense than SiO_2_, due to the large carrier density in ITO, leading to larger acoustic attenuation coefficients. Another possible reason is that ITO film could have more porosity than SiO_2_ film, resulting in more grains in ITO film, which can enhance the phonon scattering with grain boundaries^35^ and increase the acoustic attenuation coefficients.

## Conclusion

In conclusion, we demonstrated a novel technique to generate quasi-monochromatic acoustic phonons with simultaneous centre frequency and spectral bandwidth tunabilities in the GHz to sub-THz frequency range. The quasi-monochromatic acoustic phonons were generated using intensity-modulated optical pulses in a metallic transducer through the photothermal effect. The intensity-modulated optical pulses were generated via the interference of two chirped optical pulses, and the centre frequency and spectral bandwidth of the quasi-monochromatic acoustic phonons were tunable from 65 to 381 GHz and 17 to 73 GHz, respectively. A nanoscale W film was excited using the quasi-monochromatic acoustic phonon source at various frequencies, and selective excitations of two mechanical resonance modes were demonstrated. In addition, the tunable acoustic phonon source was used for frequency-dependent acoustic phonon transport measurements. The longitudinal acoustic velocities of SiO_2_ and ITO were frequency-independent in the range of 50 to 110 GHz, which are in a good agreement with the literature values. On the other hand, the longitudinal acoustic attenuation coefficients of both SiO_2_ and ITO showed quadratic frequency dependence in the range of 50 to 110 GHz, and the dominant acoustic attenuation mechanism was ascribed to be anharmonic interaction of acoustic phonons with the thermal bath. Furthermore, the acoustic attenuation coefficients in ITO were larger than SiO_2_ at certain frequencies, which was possibly related to the enhanced phonon scattering with ionised impurities and grain boundaries in ITO. The presented spectrum-tunable narrowband acoustic phonon source has potential applications in nanoscale materials characterisation, e.g., measurement of frequency-dependent acoustic attenuation in amorphous materials^26,36^, determination of ultrathin film thickness^11^, excitation of coherent phonons in quantum dots^14^, and noninvasive characterisation of mechanical properties of single cells^37^, etc.

## Methods

### Sample preparation

The sandwiched-layer structured sample was grown on a c-plane sapphire substrate with size of 10 mm by 10 mm by 0.5 mm. The sapphire substrate was washed in acetone, methanol, and isopropyl alcohol to eliminate the organic contaminants. After the cleaning procedure, the substrate was immediately blown dry using a nitrogen gun and loaded in an electron beam chamber. The chamber was pumped to be a vacuum environment with a pressure of 1 × 10^−6^ Torr. The Al receiver film, sample layer (SiO_2_ or ITO film), and Al transducer film were deposited onto the sapphire substrate using electron beam evaporation^38,39^ one after another, at a deposition rate of 0.1 nm/s, 0.2 nm/s, and 0.02 nm/s, respectively. The W film was epitaxially grown on hydrogen-terminated Si(100) surface^40,41^. The W film was deposited at a rate of 0.02 nm/s in order to enhance surface smoothness. The root-mean-square roughness of W film was measured using the atomic force microscopy, which was smaller than 1 nm.

### Spectrum-tunable narrowband acoustic phonon spectroscopy

The schematic of our experimental setup is shown in Fig. 1. A compressed laser pulse from a Ti:sapphire regenerative amplifier (Coherent Legend) with repetition rate of 1 kHz, centre wavelength of 800 nm, and pulse duration of 100 femtoseconds (fs) was used as a light source. The compressed optical pulses were sent into the pulse stretcher, and linear frequency chirps in the incident pulses were introduced by two parallel linear diffraction gratings^42^, and the amount of the chirp was controlled by the distance between the two gratings. The pulse width ${\sigma }_{n}$ of the linearly chirped pulses can be varied from 2 to 25 ps by changing the distance between two diffraction gratings using a motion-controlled delay stage. The total optical path length for the pump pulse was constant regardless of the delay position in the stretcher, hence the pump-probe timing stayed constant when the chirped pulse width was changed. The linearly chirped optical pulse was then split into two pulses by a 50/50 beam splitter, and one pulse was delayed by a variable amount of time, τ with respect to the other pulse using a Mach-Zehnder interferometer with a variable path length. The two chirped optical pulses were recombined on another 50/50 beam splitter. Since the two pulses were linearly chirped, their frequency components differed by a constant beat frequency at every moment in the time domain. The generated intensity-modulated optical pulse was further used to excite a metallic transducer to generate the acoustic phonons through the photothermal effect^16,23^. The second harmonics of the 800 nm laser pulse, which was generated by type-I beta barium borate (BBO) crystal, was used as an optical probe pulse, and the strain induced reflectivity change was detected through the photoelastic effect^16,23^. The probe pulse was delayed relative to the pump pulse in time domain by a translation stage.
